# Moderate Photoinhibition of Photosystem II Protects Photosystem I from Photodamage at Chilling Stress in Tobacco Leaves

**DOI:** 10.3389/fpls.2016.00182

**Published:** 2016-02-22

**Authors:** Wei Huang, Ying-Jie Yang, Hong Hu, Shi-Bao Zhang

**Affiliations:** ^1^Key Laboratory of Economic Plants and Biotechnology, Kunming Institute of Botany, Chinese Academy of SciencesKunming, China; ^2^Yunnan Key Laboratory for Wild Plant ResourcesKunming, China

**Keywords:** chilling temperature, electron transfer, growth light intensity, photosystem I, photosystem II, photoprotection

## Abstract

It has been indicated that photosystem I (PSI) is susceptible to chilling-light stress in tobacco leaves, but the effect of growth light intensity on chilling-induced PSI photoinhibition in tobacco is unclear. We examined the effects of chilling temperature (4°C) associated with moderate light intensity (300 μmol photons m^-2^ s^-1^) on the activities of PSI and photosystem II (PSII) in leaves from sun- and shade-grown plants of tobacco (*Nicotiana tabacum* cv. k326). The sun leaves had a higher activity of alternative electron flow than the shade leaves. After 4 h chilling treatment, the sun leaves showed significantly a higher PSI photoinhibition than the shade leaves. At chilling temperature the sun leaves showed a greater electron flow from PSII to PSI, accompanying with a lower P700 oxidation ratio. When leaves were pre-treated with lincomycin, PSII activity decreased by 42% (sun leaves) and 47% (shade leaves) after 2 h exposure to the chilling-light stress, but PSI activity remained stable during the chilling-light treatment, because the electron flow from PSII to PSI was remarkably depressed. These results indicated that the stronger chilling-induced PSI photoinhibition in the sun leaves was resulted from a greater electron flow from PSII to PSI. Furthermore, moderate PSII photoinhibition depressed electron flow to PSI and then protected PSI activity against further photodamage in chilled tobacco leaves.

## Introduction

During the winter and spring, the combination of daytime chilling temperatures and moderate light intensity are typical climatic conditions in subtropical and temperate regions. Such conditions can cause photodamage to photosystem I (PSI) in several species, including *Cucumis sativus* (Sonoike and Terashima, 1994; Terashima et al., 1994; Sonoike, 1995, 1999; Kudoh and Sonoike, 2002; Zhang et al., 2011), *Spinacia oleracea* (Sonoike, 1995; Hwang et al., 2004), *Solanum tuberosum* (Havaux and Davaud, 1994), *Arabidopsis thaliana* (Zhang and Scheller, 2004), and *Nicotiana tabacum* (Barth and Krause, 1999, 2002). The electrons supplied from PSII to PSI induce the production of superoxide anion radicals that can be converted to hydrogen peroxide (Asada, 1999). This H_2_O_2_ reacts with reduced iron in the iron-sulfur centers to form hydroxyl radicals that immediately cause damage to those centers in the PSI complex (Sonoike et al., 1997). In previous studies on chilling stress and PSI activity, all plant materials were grown under low or moderate light intensities. However, in routine production, chilling-sensitive crop plants, e.g., cucumber, potato, and tobacco, are usually cultivated under field conditions that include brighter illumination. Because photosynthetic electron flow and photoprotective mechanisms such as cyclic electron flow (CEF) and no-photochemical quenching (NPQ) are affected by growth light intensity (Miyake et al., 2005), any examination of the response of PSI activity to chilling-light stress should consider the level of growth light condition. However, little is known about the influence of growth light condition on that scenario.

At normal growing temperatures (e.g., 25°C) and low light, the electron flow from PSII does not exceed the capacity of PSI electron acceptors to cope with electrons, and PSI remains stable (Munekage et al., 2002; Tikkanen et al., 2010, 2014). Damage to PSI occurs only when this electron flow exceeds the capacity of those PSI acceptors (Tikkanen and Aro, 2014; Tikkanen et al., 2014). At a chilling temperature, inhibition of the Calvin Cycle can induce an increase in NADPH/NADP^+^, leading to the reduction in electron transport chains and the production of superoxide anion radicals (Murata et al., 2007). This subsequently causes photodamage to PSI in cucumber, spinach, and *Arabidopsis thaliana* (Sonoike, 2006). When the electron flow from PSII to PSI is blocked by DCMU and DBMIB, PSI photoinhibition is not observed in the chilled leaves of potato, cucumber and spinach, apparently because those electron transport chains are oxidized and production of superoxide anion radicals on the PSI acceptor side is inhibited (Havaux and Davaud, 1994; Sonoike, 1995). Therefore, based on those reports, one might conclude that electron flow from PSII is necessary for PSI photoinhibition at chilling temperatures.

Tobacco plants grown under high light have greater photosynthetic capacity and electron transport from PSII to PSI than those exposed to low light, regardless of the temperature at which measurements are made (Yamori et al., 2010a). Consequently, we speculated that, when illuminated at chilling temperature, tobacco leaves grown under high light had higher electron flow from PSII to PSI than those leaves grown under low light. Because the electron transport responding to alternative electron sinks contributes to the production of ROS in the acceptor side of PSI, the sensitivity of PSI to photoinhibition at chilling temperature is induced by alternative electron flow (Sonoike, 1995). However, it is unclear whether the difference of chilling-induced photoinhibition of PSI between tobacco sun and shade leaves is related to alternative electron flow.

Photoinhibition of PSII was regarded as an ultimate mechanism for protecting PSI activity in *pgr5* mutants of *Arabidopsis thaliana* that lack PGR5-dependent CEF (Tikkanen et al., 2014). When PSII activity was decreased by about 40% in *pgr5* plants, PSI activity was protected against further photodamage because of decreased electron flow from PSII (Tikkanen et al., 2014). In plants, CEF and NPQ are two protective mechanisms for PSII activity (Munekage et al., 2002, 2004; Takahashi et al., 2009; Brestic et al., 2014, 2015; Zivcak et al., 2014a). Activation of CEF and NPQ alleviate PSII photoinhibition at chilling temperature (Kim et al., 2001; Li et al., 2004; Huang et al., 2011). Tobacco plants grown under high light had greater capacities for CEF and NPQ when compared with plants grown under low light (Miyake et al., 2005). The extent of chilling-induced PSII photoinhibition is diminished in more brightly lit plants. The ROS production was highly correlated to PSII activity (Oukarroum et al., 2015). Thus, the higher PSII activity in those plants probably aggravates PSI photoinhibition under chilling-light stress.

Here, we investigated the response of PSI and PSII activities to 4°C and 300 μmol photons m^-2^ s^-1^ in tobacco leaves grown under two different light conditions (95% sunlight for sun leaves, 28% sunlight for shade leaves). Our aim was to examine whether the growth light intensity influences the response of PSI activity to combined chilling and light stresses. Here, PSI was more susceptible to such stress in sun leaves than shade leaves, due to higher electron flow from PSII to PSI. When PSII repair was inhibited by lincomycin, a large decrease in PSII activity limited electron flow from PSII to PSI, and thus PSI activity was not sensitive to chilling-light stress in either leaf type.

## Materials and Methods

### Plant Materials

Seedlings of the ‘k326’ cultivar from tobacco (*Nicotiana tabacum*) were cultivated in plastic pots in a phytotron at Kunming Institute of Botany, Yunnan, China (elevation 1900 m, 102°41′E, 25°01′N). Day/night temperatures were 24°C/18°C. Relative humidity was kept at 60% and the atmospheric CO_2_ concentration (*C*_a_) was held at 400 μmol mol^-1^. The phytotron used sunlight as the source of illumination, and the light intensity received by sun plants was about 95% of full sunlight (maximum intensity at noon ≈ 1990 μmol photons m^-2^ s^-1^). The shade plants were grown under 28% sunlight (maximum intensity ≈ 580 μmol photons m^-2^ s^-1^). During the experimental period, none of the plants experienced any water or nutrient stresses. After the plants were transplanted and cultivated for 50 days, the newly produced, mature leaves were used for photosynthetic measurements.

### Simultaneous Measurements of Chlorophyll Fluorescence and P700 Redox State

A Dual-PAM-100 system (Heinz Walz, Effeltrich, Germany) was used for simultaneous measurements of chlorophyll fluorescence and the P700 redox state. In the early morning, after dark-adaptation overnight, values for *F*_v_/*F*_m_ were obtained from intact mature leaves (*F*_v_, variable fluorescence; *F*_m_, maximum fluorescence). Those leaves with *F*_v_/*F*_m_ values > 0.8 were chosen for chilling treatments.

The following chlorophyll fluorescence parameters were calculated: *F*_o_′ = *F*_o_/[(*F*_m_ – *F*_o_)/*F*_m_ + *F*_o_/*F*_m_′] (Oxborough and Baker, 1997), qL = (*F*_m_′ – *F*_s_)/(*F*_m_′ – *F*_o_′) ×*F*_o_′/*F*_s_. *F*_o_ and *F*_m_ are the minimum and maximum fluorescence after dark-adaptation; *F*_o_′ and *F*_m_′ are the minimum and maximum fluorescence under light, respectively; qL is the coefficient of photochemical quenching based on the “lake” model (Oxborough and Baker, 1997); *F*_s_ is the light-adapted steady-state fluorescence; and Y(II) is the effective quantum yield of PSII under light. *F*_m_ and *F*_m_′ were measured upon illumination with a 300-ms pulse of saturating light (10000 μmol photons m^-2^ s^-1^). Because damage to PSI increases *F*_o_ and, therefore, *F*_v_/*F*_m_ is affected by photodamage to both PSI and PSII, we used *F*_m_ to estimate the amount of active PSII reaction centers (Tikkanen et al., 2014).

The maximum photo-oxidizable P700 was measured with a dual wavelength unit (830/875 nm) according to the method of Klughammer and Schreiber (2008). A saturation pulse (10000 μmol photons m^-2^ s^-1^) was applied for assessing P700 parameters. The P700^+^ signal (*P*) varies between a minimum (P700 fully reduced) and maximum level (P700 fully oxidized). At a defined optical property, the amplitude of *P*_m_ depends on the maximum amount of photo-oxidizable P700. As a result, the alteration in *P*_m_ serves as an indicator of change in PSI activity (Huang et al., 2010a,b, 2013; Gao and Wang, 2012; Suorsa et al., 2012). In our present study, *P*_m_ was measured to estimate the amount of PSI reaction centers. *P*_m_′ was also defined in analogy to the fluorescence parameter *F*_m_′. *P*_m_′ was determined similarly to *P*_m_, but with background actinic light instead of far-red illumination. The P700 oxidation ratio [Y(ND)] was measured as *P*/*P*_m_ (Pfundel et al., 2008; Huang et al., 2011, 2012; Suorsa et al., 2012; Tikkanen et al., 2014).

### Simultaneous Measurements of Gas Exchange and Chlorophyll Fluorescence

An open gas exchange system incorporating infrared CO_2_ and water vapor analyzers (Li-6400XT; Li-Cor Inc., Lincoln, NE, USA) was used to determine the rate of CO_2_ assimilation (*A*_n_) in the phytotron. Chlorophyll fluorescence was measured simultaneously with gas exchange measurements using a fluorometer chamber (6400-40; Li-Cor Inc.). The fluorescence parameters *F*_s_ and *F*_m_′ were determined as previously described (Baker and Rosenqvist, 2004), with *F*_s_ representing the steady fluorescence and *F*_m_′ the maximum fluorescence after light-adaptation. The effective quantum yield of PSII was calculated as Φ_PSII_ = (*F*_m_′ – *F*_s_)/*F*_m_′ (Genty et al., 1989). During the measurement period, the relative air humidity was 60% and the air temperature was 24°C. To generate a light response curve, the leaves of both sun and shade plants were exposed to high light (i.e., 1200 μmol photons m^-2^ s^-1^) for 20 min to obtain a steady state. Afterward, photosynthetic parameters were evaluated every 2 min at a controlled *C*_a_ of 400 μmol mol^-1^ and photosynthetic photon flux densities (PPFDs) of 2000, 1600, 1200, 800, 500, 300, 200, 100, 50, 20, or 0 μmol photons m^-2^ s^-1^. The PSII electron transport rate (*J*_F_) based on chlorophyll fluorescence measurement was calculated as *J*_F_ = 0.85 × 0.5 × PPFD × Φ_PSII_ (Miyake et al., 2005; Zhang et al., 2013; Huang et al., 2014, 2015). The rate of electron transport consumed by carboxylation plus oxygenation of RuBP (*J*_G_) was calculated as *J*_G_ = 4(*A*_n_ + *R*_d_)(*C*_i_ + 2Γ^∗^)/(*C*_i_ – Γ^∗^) (Harley et al., 1992; Zivcak et al., 2013), where *A*_n_ represents measured CO_2_ assimilation rate, *R*_d_ represents the mitochondrial respiration measured after 5 min dark adaptation, *C*_i_ represents the intercellular CO_2_ concentration, and Γ^∗^represents the CO_2_ compensation point measured in the absence of respiration. The value of Γ^∗^ was calculated to be 32.2 at 25°C according to Long and Bernacchi (2003).

### Photoinhibitory Treatment at 4°C

To examine the effect of growth light condition on chilling-induced PSI photoinhibition, detached sun and shade leaves incubated with water overnight in darkness were transferred to 4°C and 300 μmol photons m^-2^ s^-1^. To examine the effect of PSII photoinhibition on chilling-induced PSI photoinhibition, detached sun and shade leaves incubated in the presence of lincomycin (Lin, 1 mM) overnight in darkness were transferred to 4°C and 300 μmol photons m^-2^ s^-1^. Before chilling-light treatment, qL and Y(ND) were measured at 25°C and 297 μmol photons m^-2^ s^-1^. After chilling-light treatment for 2, 4, and 6 h, qL and Y(ND) were measured immediately at 4°C and 297 μmol photons m^-2^ s^-1^. Subsequently, *P*_m_ and *F*_m_ were measured after 30 min dark adaptation.

### Statistical Analysis

All results were displayed as mean values of six independent experiments. Data were subjected to an Independent-Samples *T*-test with SPSS 16.0 statistical software. Independent-Samples *T*-test was used at α = 0.05 significance level to determine whether significant differences existed between different treatments.

## Results

### Alternative Electron Flow in the Sun and Shade Leaves

**FIGURE 1:**
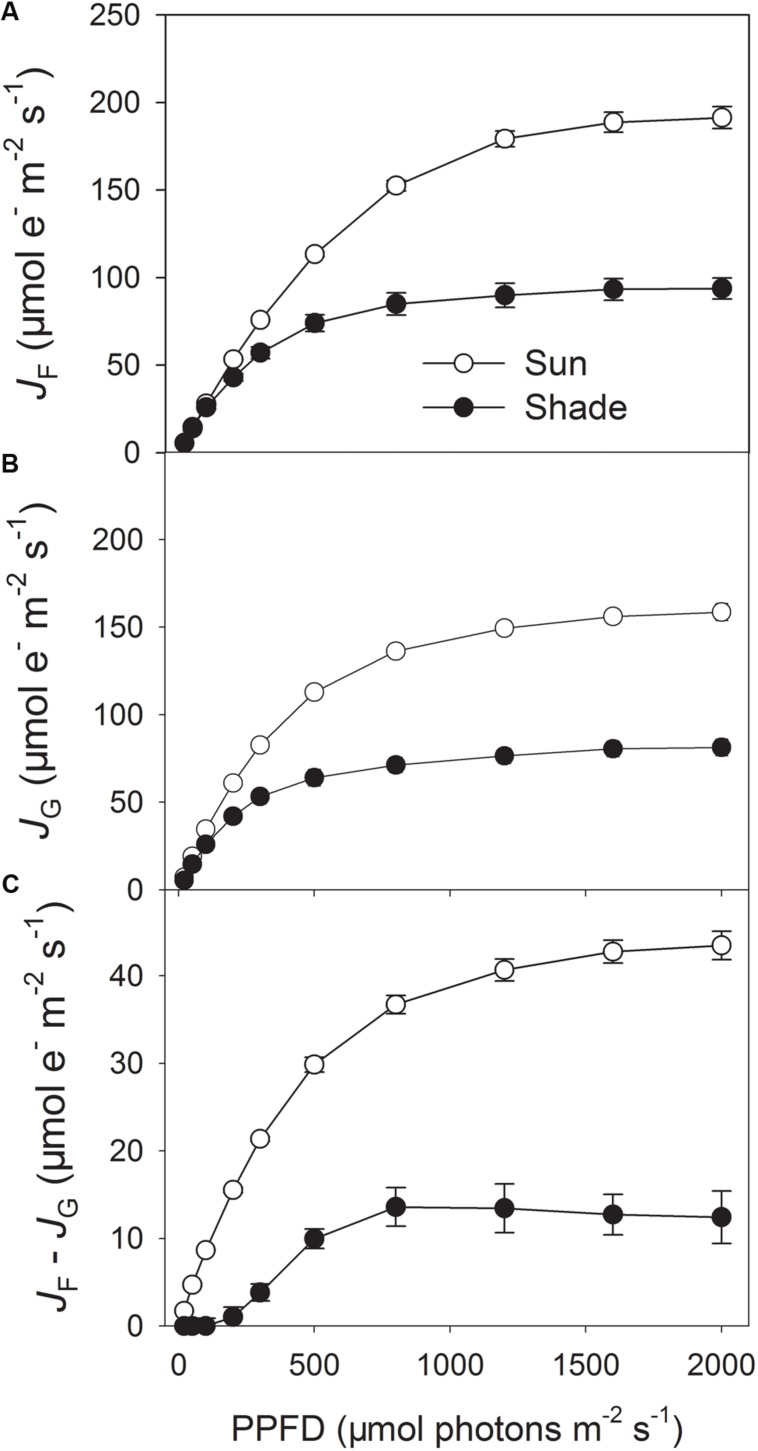
**Light response changes in photosynthetic electron flow in the sun and shade leaves of tobacco. (A)** PSII electron transport rate (*J*_F_) calculated based on measurements of PSII quantum yields, assuming the equal distribution of absorbed light between PSI and PSII. **(B)** Rate of electron transport consumed by carboxylation plus oxygenation of RuBP (*J*_G_), calculated by the data from gas exchange measurements. **(C)** The difference between *J*_F_ and *J*_G_, *J*_F_ – *J*_G_ represents a common way to estimate alternative electron flow besides the Calvin cycle and photorespiration. Values are means ± SE (*n* = 6).

To estimate the linear electron flow that is not used for RuBP carboxylation and photorespiration, PSII electron flow calculated from chlorophyll fluorescence measurements (*J*_F_) and electron transport calculated from gas exchange (*J*_G_) was compared in the sun and shade leaves (**Figures 1A,B**). The difference between *J*_F_ and *J*_G_ represents the electron flow utilized by alternative electron sinks. Light response curves indicated that under high light the sun leaves had significantly higher values of *J*_F_ and *J*_G_ at 25°C (**Figures 1A,B**), due to higher rate of CO_2_ assimilation and photorespiration (Huang et al., 2014). Furthermore, the value of *J*_F_ – *J*_G_ largely differed between the sun and shade leaves (**Figure 1C**), indicating the sun leaves had significantly a higher capacity of alternative electron flow than the shade leaves.

### Photoinhibition of PSI and PSII

**FIGURE 2:**
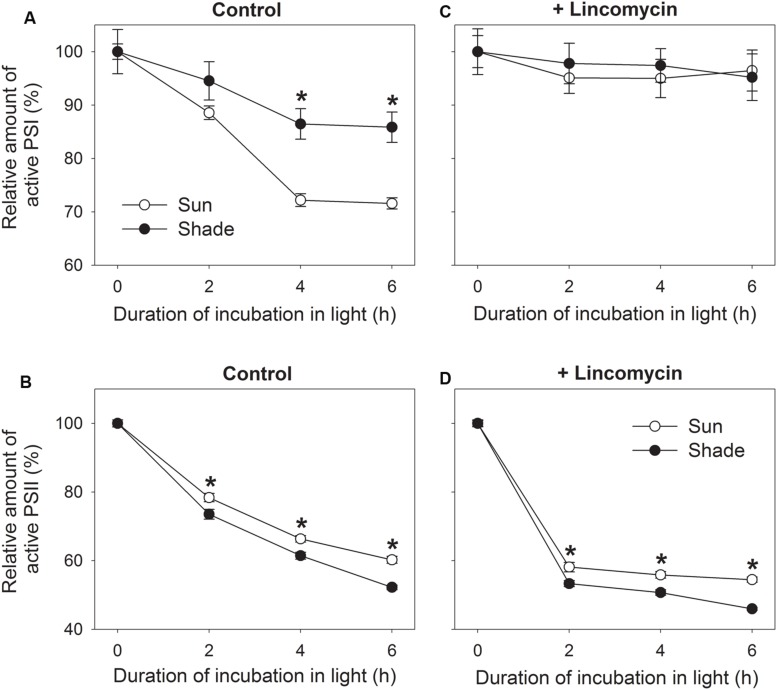
**Relationship between photoinhibition of PSII (B,D) and PSI (A,C) in sun and shade tobacco leaves.** Detached leaves incubated in the presence or absence of lincomycin (1 mM) overnight in darkness were exposed to 4°C and 300 μmol photons m^-2^ s^-1^ for 2, 4, or 6 h. *F*_m_ was measured after dark adaptation to estimate the amount of active PSII centers. *P*_m_ was measured after dark adaptation to estimate the amount of active PSI centers. All values are expressed relative to the controls before chilling-light treatment, and shown as means ± SE (*n* = 6). Asterisks indicate significant differences between the sun and shade leaves.

To examine the effect of growth light condition on chilling-induced PSI photoinhibition, detached sun and shade leaves incubated with water overnight in darkness were transferred to 4°C and 300 μmol photons m^-2^ s^-1^. By contrast, exposure for 4 h was associated with declines in *P*_m_ of 28% and 14% for sun and shade leaves, respectively (**Figure 2A**). This indicated that PSI activity was more sensitive to chilling-light stress in the sun leaves. However, prolonging the chilling period did not enhance PSI photodamage in either sun or shade leaves (**Figure 2A**).

Under such stress, shade leaves showed higher PSII photoinhibition when compared with the sun leaves. For example, after 2, 4, and 6 h of treatment, *F*_m_ values decreased by 22, 34, and 40% in the sun leaves, respectively, versus declines of 26, 39, and 48% in the shade leaves (**Figure 2B**). The extent of PSII photoinhibition differed slightly between the two types. In the initial 4 h chilling treatment, the reduction in PSII activity was accompanied by a decrease in PSI activity for both type leaves (**Figures 2A,B**). Between hours 4 and 6, PSII activity continued to drop whereas that of PSI was unaffected (**Figures 2A,B**). These results suggested that the chilling-induced decrease in PSI activity was dependent on a high PSII activity, and that PSI might have been protected from further photodamage while PSII activity declined by approximately 40%.

To further understand the effect of chilling-induced PSII photoinhibition on PSI activity, detached leaves incubated with lincomycin overnight in darkness were exposed to the above chilling-light stress. Although neither the sun nor the shade leaves showed a significant reduction in *P*_m_ values (**Figure 2C**), the *F*_m_ for both leaf types was largely decreased. For example, after exposure to the combined stress for 2, 4, and 6 h, *F*_m_ dropped by 42, 44, and 46%, respectively, in the sun leaves, and by 47, 49, and 54%, respectively, in the shade leaves (**Figure 2D**). Therefore, in the presence of lincomycin, PSII photoinhibition was aggravated and then photodamage to PSI was prevented. This strongly suggested that chilling-induced PSI photoinhibition was dependent on PSII activity.

### Relative Q_A_ Reduction and PSI Redox State

**FIGURE 3:**
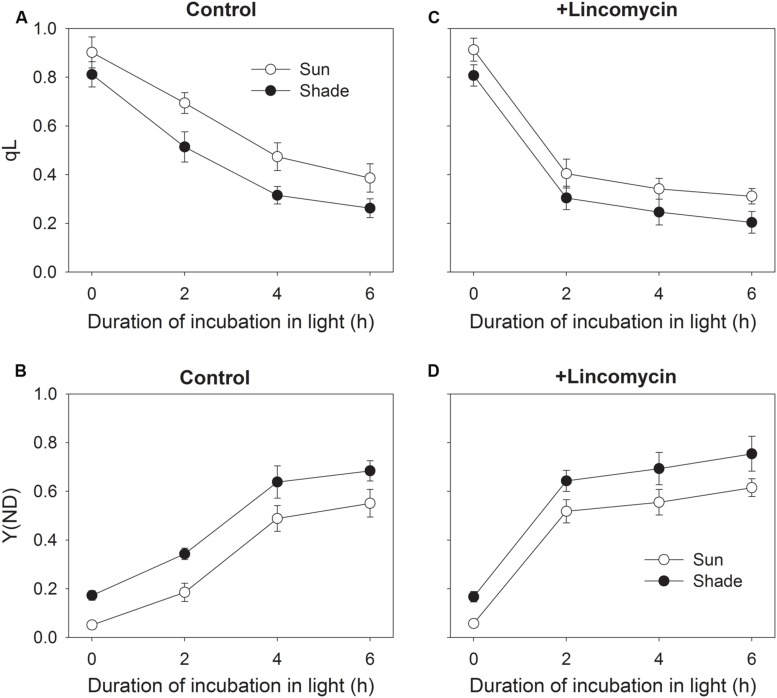
**Changes in relative Q_A_ reduction (qL) (A,C) and PSI oxidation ratio [Y(ND)] (B,D) during exposure to 4°C and 300 μmol photons m^-2^ s^-1^.** After measuring qL and Y(ND) at 24°C and 297 μmol photons ^-2^ s^-1^, leaves were incubated in the presence or absence of lincomycin (1 mM) overnight in darkness and subsequently exposed to 4°C and 300 μmol photons m^-2^ s^-1^ for 2, 4, or 6 h. During chilling-light treatment, qL and Y(ND) were measured at 4°C and 297 μmol photons m^-2^ s^-1^. Values are means ± SE (*n* = 6).

To understand the effect of chilling-induced PSII photoinhibition on relative Q_A_ reduction and PSI redox state, the changes in qL and Y(ND) during chilling treatment were measured. During chilling-light treatment, qL decreased gradually in both the sun and shade leaves (**Figure 3A**). The sun leaves showed significantly higher qL values than the shade leaves during chilling-light treatment (**Figure 3A**). This result implied that at chilling temperature the sun leaves displayed higher electron flow from PSII to PSI. With increasing time of chilling-treatment, Y(ND) gradient increased in both type leaves. Furthermore, the Y(ND) values were lower in the sun leaves compared with the shade leaves (**Figure 3B**). Interestingly, the value of Y(ND) was lower than 0.2 in the sun leaves after 2 h chilling treatment, implying the over-reduction of PSI acceptor side. In the presence of lincomycin, qL largely decreased after initial 2 h exposure to chilling-light stress in both the sun and shade leaves (**Figure 3C**). Meanwhile, Y(ND) largely increased in them (**Figure 3D**). These results indicated that, when down-regulation of PSII activity was induced by mild lincomycin treatment, the electron flow from PSII to PSI was limited, resulting in the decrease in qL and the increase in Y(ND).

## Discussion

### PSI Activity in Tobacco is More Sensitive to Chilling-Light Stress in Sun Leaves

Previous studies indicated that PSI activity was sensitive to chilling-light stress in tobacco leaves grown under low light (Barth and Krause, 1999, 2002). However, the effect of chilling-light stress on PSI activity is unclear for tobacco leaves grown under high light. Our results strongly indicated that chilling-induced PSI photoinhibition was significantly stronger in the sun leaves than the shade leaves (**Figure 2A**). This suggested that the extent of chilling-induced PSI photoinhibition was influenced by growth light intensity. The effect of growth irradiance on chilling-induced PSI photoinhibition was previously examined in common bean (Sonoike et al., 1995). Common bean leaves grown in 6.5% of full sunlight displayed stronger chilling-induced PSI photoinhibition than those grown in full sunlight (Sonoike et al., 1995). On the contrary, our results indicated that PSI was more sensitive to chilling-light stress in sun leaves than shade leaves in tobacco. Thus, we assumed that the effect of growth light condition on chilling-induced PSI photoinhibition strongly depended on plant species.

It has been indicated that, when exposed to chilling-light stress, the inhibition of Calvin cycle decreases the NADP^+^/NADPH ratio and leads to the generation of superoxide anion radicals (Murata et al., 2007), which can be converted into H_2_O_2_ (Asada, 1999). In the presence of reduced metal ions, this H_2_O_2_ is converted to the hydroxyl radical, which is highly reactive and destroys the iron–sulfur centers on the acceptor side of PSI (Sonoike, 1995, 2006, 2011). Excess electron flow from PSII to PSI can lead to reduction of PSI acceptors and production of superoxide anion radicals (Oukarroum et al., 2015), as a result, PSI only gets photodamaged when electron transfer to PSI is in excess of the capacity of PSI electron acceptors (Tikkanen and Aro, 2014; Tikkanen et al., 2014). Our results indicated that the sun leaves of tobacco had a higher capacity of alternative electron flow than the shade leaves (**Figure 1C**). The alternative electron flow is mainly caused by photoreduction of O_2_, which generates ROS at the acceptor side of PSI. During chilling-light treatment, the sun leaves had higher qL values than the shade leaves (**Figure 3A**). More electrons being transferred to PSI in the sun leaves not only led to stronger production of superoxide anion radicals in PSI acceptor side, but also increased the P700 reduction ratio. Taking together, the higher PSI photoinhibition in the sun leaves was significantly related to the higher alternative electron flow at chilling-light stress.

In plants, CEF can protect PSI against photodamage under high light by preventing over-reduction on the acceptor side in PSI (Munekage et al., 2002, 2004; Suorsa et al., 2012; Tikkanen et al., 2014; Zivcak et al., 2014a; Brestic et al., 2015). At a chilling temperature, CEF alleviates PSI photoinhibition in cucumber (Kim et al., 2001; Bukhov et al., 2004) and tropical tree species (Huang et al., 2011). The capacity for CEF can also be affected by the growth light intensity to which tobacco plants are exposed, and the tobacco sun leaves have higher CEF capacity than the shade leaves (Miyake et al., 2005; Huang et al., 2015). If CEF in tobacco did in fact have a major role in protecting PSI activity against photodamage at chilling-light stress, then we would expect the sun leaves to have less PSI photoinhibition. On the contrary, the sun leaves showed significantly stronger PSI photoinhibition, indicating that CEF provided only minimal photoprotection for PSI in tobacco leaves at chilling-light stress. The slight difference in chilling-induced PSII photoinhibition between the sun and shade leaves further indicated that CEF was hardly activated at chilling temperature in tobacco leaves. Barth and Krause (2002) indicated that NAD(P)H dehydrogenase (NDH)-mediated CEF did not protect PSI against short chilling-light stress. Furthermore, NDH-dependent CEF was less important under chilling-stressed condition (Wang et al., 2006). Thus, CEF hardly prevented PSI photoinhibition in tobacco leaves illuminated at chilling temperature.

### Moderate PSII Photoinhibition Prevents PSI Photoinhibition Under Chilling-Light Stress

Our results clearly demonstrate that chilling-induced photoinhibition of PSI in tobacco leaves is dependent upon PSII activity. In the absence of lincomycin, PSI activity decreased during the first 4 h of chilling treatment (**Figure 2A**). However, longer exposure to chilling-light stress did not aggravate PSI photoinhibition in either sun or shade leaves (**Figure 2A**). After 4 h of treatment, PSII activity decreased by 34 and 39% in sun and shade leaves, respectively. Meanwhile, qL largely decreased in both the sun and shade leaves (**Figure 3A**). This large decrease in qL led to a decline in electron transfer from PSII to PSI, and then increase P700 oxidation ratio. Consequently, there was no significant decrease in PSI activity between 4 and 6 h of chilling-light treatment in both type leaves. In the presence of lincomycin, PSII activity decreased by 42 and 47% after 2 h in stressed sun and shade leaves, respectively. This large decrease in PSII activity led to a depression of linear electron flow, as indicated by the decrease in qL and increase in Y(ND) (**Figures 3C,D**). Moreover, PSI activity was maintained stable in either leaf type after 6 h chilling-light treatment in the presence of lincomycin. Therefore, when PSII activity decreased by about 40%, PSI activity was protected from further chilling-induced photodamage.

Photosystem I becomes irreversibly photodamaged if the electrons supplied from PSII to PSI exceed the capacity of PSI electron acceptors. At normal growing temperatures and low light, the electrons transported from PSII to PSI can be efficiently quenched by the Calvin cycle and photorespiratory pathway. However, when plants are subjected to a chilling temperature, the electrons transported to PSI cannot be efficiently quenched through the Calvin cycle and photorespiratory pathway, which then leads to a reduction in photosynthetic electron chains and the production of superoxide anion radicals. In other chilling-sensitive species, such as cucumber and *Arabidopsis thaliana*, chilling-light stress induces a slight decrease in PSII activity but a large decrease in PSI activity (Sonoike, 1995; Zhang and Scheller, 2004), indicating that the slight decrease in PSII activity has little influence on electron flow from PSII to PSI. The CEF-deficient *pgr5* plants showed large PSI photoinhibition upon shift to high light. However, when the PSII repair was inhibited by lincomycin in *pgr5* plants, moderate PSII photoinhibition led to a depression of linear electron flow and then protected PSI against further photodamage in *pgr5* plants (Tikkanen et al., 2014). In the samples pre-treated with lincomycin, the chilling-light stress did not cause significant PSI photoinhibition in either sun or shade leaves. Meanwhile, the Lin-treated samples had significantly lower qL and higher Y(ND) than the H_2_O-treated samples in the initial 2 h exposure to the chilling-light stress. This depression of electron flow from PSII to PSI following a decline in PSII activity increased the level of P700 oxidation and diminished the production of superoxide anion radicals (Tikkanen et al., 2014; Oukarroum et al., 2015). Taken together, our data support the proposal that moderate down-regulation of PSII has the potential role in protecting PSI activity against further photodamage at chilling-light stress.

## Conclusion

Plants regulate photosynthetic machinery to acclimate different growth conditions including changes in irradiance (Yamori et al., 2010a; Huang et al., 2014; Zivcak et al., 2014b), nutrients (Hikosaka, 1996; Kalaji et al., 2014), temperature (Yamori et al., 2010b, 2011), and water availability (Lehtimaki et al., 2010). The shade leaves have a high capacity of light reactions as compared to the capacity of the sink. Because of this, chilling-light treatment is able to induce higher lumenal protonation in the shade leaves than the sun leaves, resulting in slow-down of cytochrome *b*_6_/*f* in the shade leaves (Tikkanen and Aro, 2014). The sun leaves had higher alternative electron flow than the shade leaves. Furthermore, the higher qL values at chilling-light stress indicated higher alternative electron sinks such as photoreduction of O_2_ that produces O_2_^-^, causing stronger PSI photoinhibition in the sun leaves. Furthermore, the lower connectivity between PSII units in the shade leaves limited electron transport between PSII and PSI (Zivcak et al., 2014a), which alleviated PSI photoinhibition at chilling-light stress. When PSII photoinhibition was aggravated by the addition of lincomycin, PSI activity was insusceptible to chilling-light stress in both sun and shade leaf types, as a result of lower qL values and higher Y(ND) values. Therefore, moderate PSII photoinhibition depressed the electron flow from PSII to PSI and thus alleviated PSI photoinhibition. Our results strongly supported the hypothesis that photoinhibition of PSI occurs only when electron flow to PSI exceeds the capacity of PSI electron acceptors as proposed by recent studies (Suorsa et al., 2012; Tikkanen and Aro, 2014; Tikkanen et al., 2014). Because of the importance of PSI in photosynthetic regulation, when tobacco sun leaves are exposed to long-term chilling-light stress, a strong irreversible photodamage of PSI can lead to severe photoinhibition of PSII and finally to the death of the plant. During the short-term chilling treatment, PSII photoinhibition can be regarded as an important mechanism protecting PSI against further photoinhibition in tobacco.

## Author Contributions

WH and S-BZ conceived and designed research. WH and Y-JY conducted experiments. WH contributed new reagents or analytical tools. WH, Y-JY, and S-BZ analyzed data. WH, Y-JY, S-BZ, and HH wrote the manuscript.

## Conflict of Interest Statement

The authors declare that the research was conducted in the absence of any commercial or financial relationships that could be construed as a potential conflict of interest.

## References

[B1] Asada K. (1999). The water-water cycle in chloroplasts: scavenging of active oxygens and dissipation of excess photons. *Annu. Rev. Plant Biol.* 50 601–639. 10.1146/annurev.arplant.50.1.601 15012221

[B2] Baker N. R. Rosenqvist E. (2004). Applications of chlorophyll fluorescence can improve crop production strategies: an examination of future possibilities. *J. Exp. Bot.* 55 1607–1621. 10.1093/jxb/erh196 15258166

[B3] Barth C. Krause G. H. (1999). Inhibition of photosystem I and II in chilling-sensitive and chilling-tolerant plants under light and low-temperature stress. *Z. Naturforsch.* 54c, 645–657.

[B4] Barth C. Krause G. H. (2002). Study of tobacco transformants to assess the role of chloroplastic NAD(P)H dehydrogenase in photoprotection of photosystems I and II. *Planta* 216 273–279. 10.1007/s00425-002-0843-0 12447541

[B5] Brestic M. Zivcak M. Kunderlikova K. Sytar O. Shao H.-B. Kalaji H. M. (2015). Low PSI content limits the photoprotection of PSI and PSII in early growth stages of chlorophyll b-deficient wheat mutant lines. *Photosynth. Res.* 125 151–166. 10.1007/s11120-015-0093-1 25648638

[B6] Brestic M. Zivcak M. Olsovska K. Shao H.-B. Kalaji H. M. Allakhverdiev S. I. (2014). Reduced glutamine synthetase activity plays a role in control of photosynthetic responses to high light in barley leaves. *Plant Physiol. Biochem.* 81 74–83. 10.1016/j.plaphy.2014.01.002 24491798

[B7] Bukhov N. G. Govindachary S. Rajagopal S. Joly D. Carpentier R. (2004). Enhanced rates of P700+ dark-reduction in leaves of *Cucumis sativus* L. photoinhibited at chilling temperature. *Planta* 218 852–861. 10.1007/s00425-003-1165-6 14685857

[B8] Gao S. Wang G. C. (2012). The enhancement of cyclic electron flow around photosystem I improves the recovery of severely desiccated *Porphyra* yezoensis (Bangiales, Rhodophyta). *J. Exp. Bot.* 63 4349–4358. 10.1093/jxb/ers082 22438301

[B9] Genty B. Briantais J. M. Baker N. R. (1989). The relationship between the quantum yield of photosynthetic electron transport and quenching of chlorophyll fluorescence. *Biochim. Biophys. Acta* 990 87–92. 10.1016/S0304-4165(89)80016-9

[B10] Harley P. C. Loreto F. Marco G. D. Sharkey T. D. (1992). Theoretical considerations when estimating the mesophyll conductance to CO_2_ flux by analysis of the response of photosynthesis to CO_2_. *Plant Physiol.* 98 1429–1436. 16668811 10.1104/pp.98.4.1429 PMC1080368

[B11] Havaux M. Davaud A. (1994). Photoinhibition of photosynthesis in chilled potato leaves is not correlated with a loss of photosystem II activity – preferential inactivation of photosystem I. *Photosynth. Res.* 40 75–92. 10.1007/BF00019047 24311216

[B12] Hikosaka K. (1996). Effects of leaf age, nitrogen nutrition and photon flux density on the organization of the photosynthetic apparatus in leaves of a vine (*Ipomoea tricolor* Cav.) grown horizontally to avoid mutual shading of leaves. *Planta* 198 144–150. 10.1007/BF00197597 28313732

[B13] Huang W. Fu P. L. Jiang Y. J. Zhang J. L. Zhang S. B. Hu H. (2013). Differences in the responses of photosystem I and photosystem II of three tree species *Cleistanthus sumatranus*, *Celtis philippensis* and *Pistacia weinmannifolia* submitted to a prolonged drought in a tropical limestone forest. *Tree Physiol.* 33 211–220. 10.1093/treephys/tps132 23329334

[B14] Huang W. Yang S. J. Zhang S. B. Zhang J. L. Cao K. F. (2012). Cyclic electron flow plays an important role in photoprotection for the resurrection plant *Paraboea rufescens* under drought stress. *Planta* 235 819–828. 10.1007/s00425-011-1544-3 22080919

[B15] Huang W. Yang Y. J. Hu H. Zhang S. B. (2015). Different roles of cyclic electron flow around photosystem I under sub-saturating and saturating light intensities in tobacco leaves. *Front. Plant Sci.* 6:923 10.3389/fpls.2015.00923 PMC4621282 26579169

[B16] Huang W. Zhang S. B. Cao K. F. (2010a). The different effects of chilling stress under moderate illumination on photosystem II compared with photosystem I and subsequent recovery in tropical tree species. *Photosynth. Res.* 103 175–182. 10.1007/s11120-010-9539-7 20221850

[B17] Huang W. Zhang S. B. Cao K. F. (2010b). Stimulation of cyclic electron flow during recovery after chilling-induced photoinhibition of PSII. *Plant Cell Physiol.* 51 1922–1928. 10.1093/pcp/pcq144 20861006

[B18] Huang W. Zhang S. B. Cao K. F. (2011). Cyclic electron flow plays an important role in photoprotection of tropical trees illuminated at temporal chilling temperature. *Plant Cell Physiol.* 52 297–305. 10.1093/pcp/pcq166 21062868

[B19] Huang W. Zhang S. B. Hu H. (2014). Sun leaves up-regulate the photorespiratory pathway to maintain a high rate of CO_2_ assimilation in tobacco. *Front. Plant Sci.* 5:688 10.3389/fpls.2014.00688 PMC4253947 25520735

[B20] Hwang H. J. Kim J. H. Eu Y. J. Moon B. Y. Cho S. H. Lee C. H. (2004). Photoinhibition of photosystem I is accelerated by dimethyldithiocarbamate, an inhibitor of superoxide dismutase, during light-chilling of spinach leaves. *J. Photochem. Photobiol. B Biol.* 73 79–85. 10.1016/j.jphotobiol.2003.09.008 14732254

[B21] Kalaji H. M. Oukarroum A. Alexandrov V. Kouzmanova M. Brestic M. Zivcak M. (2014). Identification of nutrient deficiency in maize and tomato plants by in vivo chlorophyll a fluorescence measurements. *Plant Physiol. Biochem.* 81 16–25. 10.1016/j.plaphy.2014.03.029 24811616

[B22] Kim S. J. Lee C. H. Hope A. B. Chow W. S. (2001). Inhibition of photosystems I and II and enhanced back flow of photosystem I electrons in cucumber leaf discs chilled in the light. *Plant Cell Physiol.* 42 842–848. 10.1093/pcp/pce109 11522910

[B23] Klughammer C. Schreiber U. (2008). Saturation pulse method for assessment of energy conversion in PSI. *PAM Appl. Notes* 1 11–14.

[B24] Kudoh H. Sonoike K. (2002). Irreversible damage to photosystem I by chilling in the light: cause of the degradation of chlorophyll after returning to normal growth temperature. *Planta* 215 541–548. 12172835 10.1007/s00425-002-0790-9

[B25] Lehtimaki N. Lintala M. Allahverdiyeva Y. Aro E. M. Mulo P. (2010). Drought stress-induced upregulation of components involved in ferredoxin-dependent cyclic electron transfer. *J. Plant Physiol.* 167 1018–1022. 10.1016/j.jplph.2010.02.006 20392519

[B26] Li X. G. Duan W. Meng Q. W. Zou Q. Zhao S. J. (2004). The function of chloroplastic NAD(P)H dehydrogenase in tobacco during chilling stress under low irradiance. *Plant Cell Physiol.* 45 103–108. 10.1093/pcp/pch011 14749491

[B27] Long S. P. Bernacchi C. J. (2003). Gas exchange measurements, what can they tell us about the underlying limitations to photosynthesis? Procedures and sources of error. *J. Exp. Bot.* 54 2393–2401. 10.1093/jxb/erg262 14512377

[B28] Miyake C. Horiguchi S. Makino A. Shinzaki Y. Yamamoto H. Tomizawa K. (2005). Effects of light intensity on cyclic electron flow around PSI and its relationship to non-photochemical quenching of chl fluorescence in tobacco leaves. *Plant Cell Physiol.* 46 1819–1830. 10.1093/pcp/pci197 16143595

[B29] Munekage Y. Hashimoto M. Miyake C. Tomizawa K. I. Endo T. Tasaka M. (2004). Cyclic electron flow around photosystem I is essential for photosynthesis. *Nature* 429 579–582. 10.1038/nature02598 15175756

[B30] Munekage Y. Hojo M. Meurer J. Endo T. Tasaka M. Shikanai T. (2002). PGR5 is involved in cyclic electron flow around photosystem I and is essential for photoprotection in *Arabidopsis*. *Cell* 110 361–371. 10.1016/S0092-8674(02)00867-X 12176323

[B31] Murata N. Takahashi S. Nishiyama Y. Allakhverdiev S. I. (2007). Photoinhibition of photosystem II under environmental stress. *Biochim. Biophys. Acta* 1767 414–421. 10.1016/j.bbabio.2006.11.019 17207454

[B32] Oukarroum A. Bussotti F. Goltsev V. Kalaji H. M. (2015). Correlation between reactive oxygen species production and photochemistry of photosystems I and II in *Lemna gibba* L. plants under salt stress. *Environ. Exp. Bot.* 109 80–88. 10.1016/j.envexpbot.2014.08.005

[B33] Oxborough K. Baker N. R. (1997). Resolving chlorophyll a fluorescence images of photosynthetic efficiency into photochemical and non-photochemical components – calculation of qP and Fv/Fm without measuring Fo. *Photosynth. Res.* 54 135–142. 10.1023/A:1005936823310

[B34] Pfundel E. Klughammer C. Schreiber U. (2008). Monitoring the effects of reduced PS II antenna size on quantum yields of photosystems I and II using the Dual-PAM-100 measuring system. *PAM Appl. Notes* 1 21–24.

[B35] Sonoike K. (1995). Selective photoinhibition of photosystem I in isolated thylakoid membranes from cucumber and spinach. *Plant Cell Physiol.* 36 825–830.

[B36] Sonoike K. (1999). The different roles of chilling temperatures in the photoinhibition of photosystem I and photosystem II. *J. Photochem. Photobiol. B Biol.* 48 136–141. 10.1016/S1011-1344(99)00030-5

[B37] Sonoike K. (2006). “Photoinhibition and protection of photosystem I,” in *Photosystem I: The Light-Driven Plastocyanin: Ferredoxin Oxidoreductase*, ed. Golbeck J. H. (Dordrecht: Springer), 657–668.

[B38] Sonoike K. (2011). Photoinhibition of photosystem I. *Physiol. Plant.* 142 56–64. 10.1111/j.1399-3054.2010.01437.x 21128947

[B39] Sonoike K. Ishibashi M. Watanabe A. (1995). “Chilling sensitive steps in leaves of *Phaseolus vulgaris* L. Examination of the effects of growth irradiances on PSI photoinhibition,” in *Photosynthesis: From Light to Biosphere*, ed. Mathis P. (Dordrecht: Kluwer Academic Publishers), 853–856.

[B40] Sonoike K. Kamo M. Hihara Y. Hiyama T. Enami I. (1997). The mechanism of the degradation of psaB gene product, one of the photosynthetic reaction center subunits of photosystem I, upon photoinhibition. *Photosynth. Res.* 53 55–63. 10.1023/A:1005852330671

[B41] Sonoike K. Terashima I. (1994). Mechanism of photosystem-I photoinhibition in leaves of *Cucumis sativus* L. *Planta* 194 287–293. 10.1007/BF01101690

[B42] Suorsa M. Jarvi S. Grieco M. Nurmi M. Pietrzykowska M. Rantala M. (2012). Proton gradient regulation5 is essential for proper acclimation of *Arabidopsis* photosystem I to naturally and artificially fluctuating light conditions. *Plant Cell* 24 2934–2948. 10.1105/tpc.112.097162 22822205 PMC3426124

[B43] Takahashi S. Milward S. E. Fan D. Y. Chow W. S. Badger M. R. (2009). How does cyclic electron flow alleviate photoinhibition in *Arabidopsis*? *Plant Physiol.* 149 1560–1567. 10.1104/pp.108.134122 19118124 PMC2649389

[B44] Terashima I. Funayama S. Sonoike K. (1994). The site of photoinhibition in leaves of *Cucumis sativus* L. at low temperatures is photosystem I, not photosystem II. *Planta* 193 300–306. 10.1007/BF00192544

[B45] Tikkanen M. Aro E. M. (2014). Integrative regulatory network of plant thylakoid energy transduction. *Trends Plant Sci.* 19 10–17. 10.1016/j.tplants.2013.09.003 24120261

[B46] Tikkanen M. Grieco M. Kangasjarvi S. Aro E. M. (2010). Thylakoid protein phosphorylation in higher plant chloroplasts optimizes electron transfer under fluctuating light. *Plant Physiol.* 152 723–735. 10.1104/pp.109.150250 19965965 PMC2815896

[B47] Tikkanen M. Mekala N. R. Aro E. M. (2014). Photosystem II photoinhibition-repair cycle protects photosystem I from irreversible damage. *Biochim. Biophys. Acta* 1837 210–215. 10.1016/j.bbabio.2013.10.001 24161359

[B48] Wang P. Duan W. Takabayashi A. Endo T. Shikanai T. Ye J. Y. (2006). Chloroplastic NAD(P)H dehydrogenase in tobacco leaves functions in alleviation of oxidative damage caused by temperature stress. *Plant Physiol.* 141 465–474. 10.1104/pp.105.070490 16428601 PMC1475475

[B49] Yamori W. Evans J. R. von Caemmerer S. (2010a). Effects of growth and measurement light intensities on temperature dependence of CO_2_ assimilation rate in tobacco leaves. *Plant Cell Environ.* 33 332–343. 10.1111/j.1365-3040.2009.02067.x 19895395

[B50] Yamori W. Noguchi K. Hikosaka K. Terashima I. (2010b). Phenotypic plasticity in photosynthetic temperature acclimation among crop species with different cold tolerances. *Plant Physiol.* 152 388–399. 10.1104/pp.109.145862 19880611 PMC2799372

[B51] Yamori W. Sakata N. Suzuki Y. Shikanai T. Maniko A. (2011). Cyclic electron flow around photosystem I via chloroplast NAD(P)H dehydrogenase (NDH) complex performs a significant physiological role during photosynthesis and plant growth at low temperature in rice. *Plant J.* 68 966–976. 10.1111/j.1365-313X.2011.04747.x 21848656

[B52] Zhang S. P. Scheller H. V. (2004). Photoinhibition of photosystem I at chilling temperature and subsequent recovery in *Arabidopsis*. *Plant Cell Physiol.* 45 1595–1602. 10.1093/pcp/pch180 15574835

[B53] Zhang W. Huang W. Yang Q. Y. Zhang S. B. Hu H. (2013). Effect of growth temperature on the electron flow for photorespiration in leaves of tobacco grown in the field. *Physiol. Plant.* 149 141–150. 10.1111/ppl.12044 23480306

[B54] Zhang Z. Jia Y. Gao H. Zhang L. Li H. Meng Q. (2011). Characterization of PSI recovery after chilling-induced photoinhibition in cucumber (*Cucumis sativus* L.) leaves. *Planta* 234 883–889. 10.1007/s00425-011-1447-3 21647604

[B55] Zivcak M. Brestic M. Balatova Z. Drevenakova P. Olsovska K. Kalaji H. M. (2013). Photosynthetic electron transport and specific photoprotective responses in wheat leaves under drought stress. *Photosynth. Res.* 117 529–546. 10.1007/s11120-013-9885-3 23860828

[B57] Zivcak M. Brestic M. Kalaji H. M. Govindjee (2014b). Photosynthetic responses of sun- and shade-grown barley leaves to high light: is the lower PSII connectivity in shade leaves associated with protection against excess of light? *Photosynth. Res.* 119 339–354. 10.1007/s11120-014-9969-8 24445618 PMC3923118

[B56] Zivcak M. Kalaji H. M. Shao H.-B. Olsovska K. Brestic M. (2014a). Photosynthetic proton and electron transport in wheat leaves under prolonged moderate drought stress. *J. Photochem. Photobiol. B Biol.* 137 107–115. 10.1016/j.jphotobiol.2014.01.007 24508481

